# Recent updates in applications of nanomedicine for the treatment of hepatic fibrosis

**DOI:** 10.3762/bjnano.15.89

**Published:** 2024-08-23

**Authors:** Damai Ria Setyawati, Fransiska Christydira Sekaringtyas, Riyona Desvy Pratiwi, A’liyatur Rosyidah, Rohimmahtunnissa Azhar, Nunik Gustini, Gita Syahputra, Idah Rosidah, Etik Mardliyati, Tarwadi, Sjaikhurrizal El Muttaqien

**Affiliations:** 1 Research Center for Vaccine and Drugs, National Research and Innovation Agency (BRIN), LAPTIAB 1, PUSPIPTEK, Tangerang Selatan 15314, Indonesia; 2 Research Center for Pharmaceutical Ingredients and Traditional Medicine, National Research and Innovation Agency (BRIN), LAPTIAB 1, PUSPIPTEK, Tangerang Selatan 15314, Indonesia

**Keywords:** active targeting, hepatic fibrosis, nanocarriers, nanomedicine, passive targeting

## Abstract

Over recent decades, nanomedicine has played an important role in the enhancement of therapeutic outcomes compared to those of conventional therapy. At the same time, nanoparticle drug delivery systems offer a significant reduction in side effects of treatments by lowering the off-target biodistribution of the active pharmaceutical ingredients. Cancer nanomedicine represents the most extensively studied nanotechnology application in the field of pharmaceutics and pharmacology since the first nanodrug for cancer treatment, liposomal doxorubicin (Doxil^®^), has been approved by the FDA. The advancement of cancer nanomedicine and its enormous technological success also included various other target diseases, including hepatic fibrosis. This confirms the versatility of nanomedicine for improving therapeutic activity. In this review, we summarize recent updates of nanomedicine platforms for improving therapeutic efficacy regarding liver fibrosis. We first emphasize the challenges of conventional drugs for penetrating the biological barriers of the liver. After that, we highlight design principles of nanocarriers for achieving improved drug delivery of antifibrosis drugs through passive and active targeting strategies.

## Introduction

**Figure 1:**
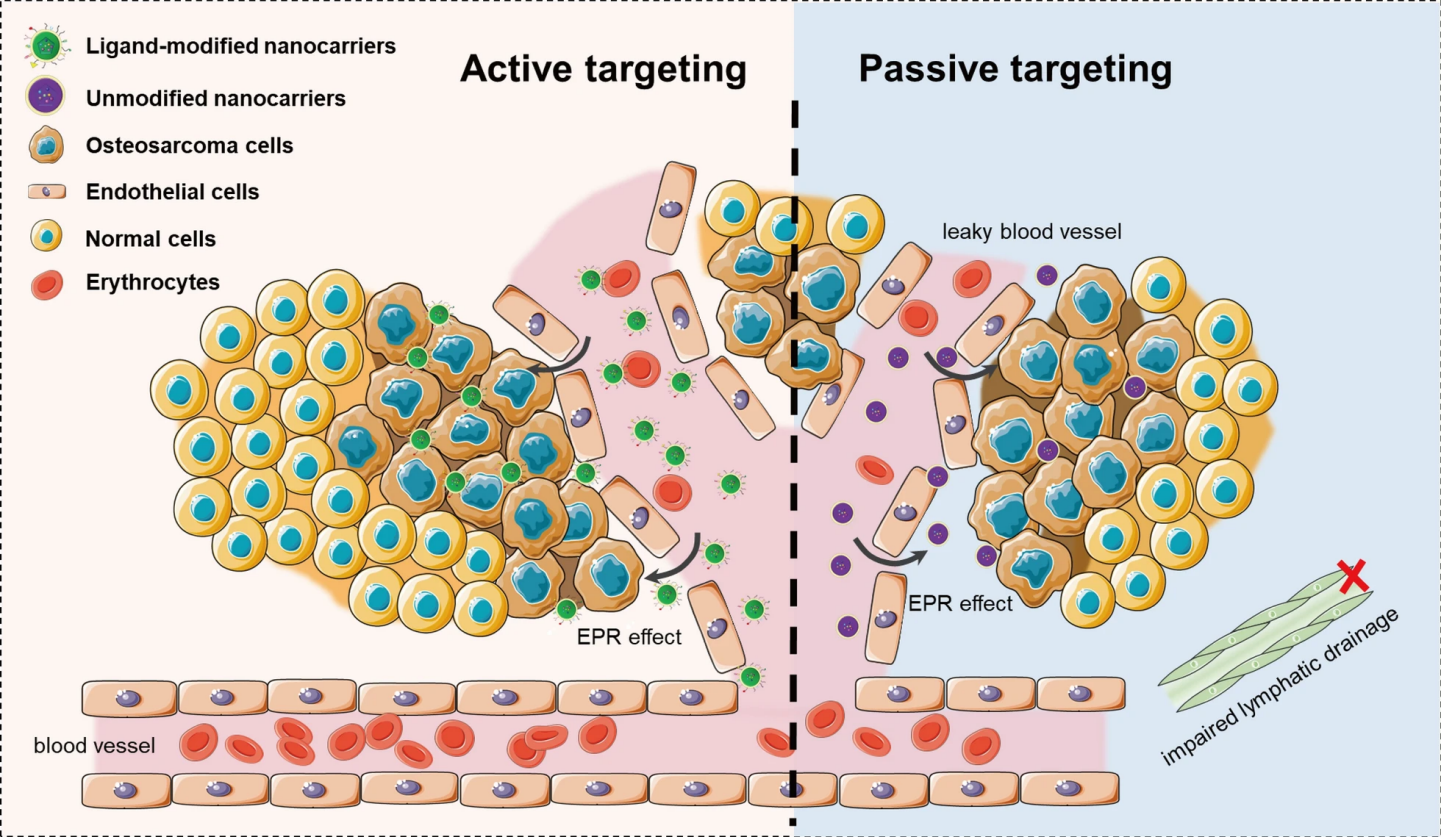
Schematic illustration of the passive accumulation of NPs through the EPR effect (right panel) and of actively targeting overexpressed markers in cancer cells (left panel). The right panel displays leaky vasculature of tumor vessels with lack of effective lymphatic drainage, allowing for a higher permeability of macromolecular drugs and higher retention. Figure 1 was reproduced from [7] (© 2023 P. Shi et al., published by BMC, distributed under the terms of the Creative Commons Attribution 4.0 International License, https://creativecommons.org/licenses/by/4.0).

Over the last three decades, we have witnessed tremendous progress in the field of nanomedicine through the preparation of a vast number of nanoscale (bio)materials. Nanomedicine itself is defined as the biomedical application of nanoscale systems with unique physicochemical properties, including small size, large specific surface area, high reactivity, and quantum effects of the nanoparticles (NPs) [1–2]. Nanomedicine is specifically designated for therapeutics (drug delivery), diagnostics, and imaging, as well as for regenerative medicine. Aiming to improve the treatment outcomes, new nanomedicinal drugs and formulations have been reported on an almost daily basis for targeting various diseases. Until now, most nanomedicine applications have focused primarily on drug delivery and theranostic nanoplatforms for cancer treatment. The enhanced permeability and retention (EPR) effect, first described by Maeda and co-workers in 1986, allows for high accumulation of the drug nanocarriers via the leaky vasculature and the deficient lymphatic system around solid tumors, as illustrated in the right panel of Figure 1 [3–5]. The EPR effect has been a cornerstone for cancer nanomedicine development, and various types of nanocarrier drug delivery systems have been developed to take advantage of this passively targeted strategy. Moreover, active targeting strategies have been developed to further improve the drug accumulation selectively through specific binding to receptors overexpressed by cancer cells (left panel of Figure 1), resulting in enhanced therapeutic activity and reduced systemic toxicity. Globally, there are around 15 approved cancer drug nanoformulations for clinical use, and 80 candidates for novel cancer nanomedicines are now under evaluation in clinical stages [6]. Simple liposomal and micellar formulations containing chemotherapeutic agents still predominate in this group.

Despite the obstacles and challenges, oncology has become the main focus of nanomedicine [8]. Liposomal doxorubicin was the first FDA-approved nanodrug (1995) achieving improved therapeutic efficacy through passive targeting via the EPR effect [9]. The clinical applications of nanomedicine then shifted to other diseases, legitimating nanomedicine as a strategy to increase the therapeutic activity. This is supported by the fact that there are over 50 nanotechnology-based medical products approved by regulatory bodies worldwide for various medical purposes, including AmBisome^®^ (liposomal amphotericin B) for fungal infections, Visudyn^®^ (liposomal vertepor) for macular degeneration, and Onpattro^®^ (lipid nanoparticles with small interfering RNA) for hereditary transthyretin amyloidosis (ATTR) [10]. Here, we describe the mechanism of nanomedicine-based drug delivery for liver fibrosis treatment.

In the following review, we briefly summarize the basic physiology of liver fibrosis, the interaction between NPs and the liver, and the corresponding relationships regarding passive and active liver targeting strategies. We then highlight updates from the last five years regarding liver fibrosis targeting using various types of therapeutic compounds and a wide range of nanocarriers, including polymeric NPs, solid lipid NPs and their derivatives, and inorganic NPs. We also discuss the underlying mechanisms of some nanocarriers to yield selective liver accumulation and enhance therapeutic action. In the final section, we mention the future perspective of the development of nanomedicine-based drug delivery for liver fibrosis.

## Review

### Liver fibrosis

Among hepatic diseases, liver fibrosis has become a major global health burden. It accounts for approximately two million deaths per year worldwide with no clinically approved pharmacotherapy [11]. This disease is characterized by abnormal physiological constitution of the liver due to the trans-differentiation of hepatic stellate cells (HSCs) into collagen-producing myofibroblasts, resulting in the progressive accumulation of extracellular matrix (ECM) protein [12]. The condition may be caused by various etiologies, including viral hepatitis infection, alcohol abuse, and metabolic-associated fatty liver disease [13]. Currently, numerous therapeutic strategies are under development. The antifibrotic strategies either target HSCs or use non-HSC antifibrotic targets. The non-HSC-mediated therapies focus on anti-inflammatory approaches, including the removal of the cause of parenchymal tissue injury and attenuating parenchymal stress and inflammation. In this approach, anti-inflammatory substances are used, such as corticosteroids, colchicine, and ursodeoxycholic acid [14–15]. As the activation of HSCs is a hallmark of liver ﬁbrosis, targeting signaling molecules involved in the activation of HSCs is the most important strategy in liver ﬁbrosis therapy. It includes both inhibition of HSC proliferation and of pro-fibrogenic cytokine and growth factor secretion.

In the last few years, the research on interferon γ (IFNγ), the angiotensin II-receptor antagonist Losartan, interleukin 10 (IL10), and simtuzumab showed promising antifibrosis results [16–19]. However, most of them displayed inadequate therapeutic efﬁcacies, and their use was often accompanied with unwanted side effects, resulting in unsuccessful clinical trials. This may be due to the inability of the conventional delivery platform to deliver a minimum concentration of these therapeutic molecules into the liver, as well as the lack of specificity. Without any targeting strategy, the potent antifibrotic activity of IFNγ, for example, was offset by its proinflammatory effects on macrophages [20]. Therefore, liver-targeted nanocarriers are needed to increase the drug concentration in the liver with minimum off-target effects.

For this purpose, both passive and active targeting strategies of nanomedicine-based drug deliveries have been studied. Liposomes, micelles, solid lipid NPs, and gold NPs are examples of nanoparticulates researched regarding liver fibrosis treatment. These nanocarriers allow for efficient containment of the antifibrotic compounds, particularly those with poor water solubility and low bioavailability. In addition, they protect the drug from unwanted metabolism and may facilitate penetration through biological barriers, leading to the alteration of the drug’s pharmacological activity. Among them, lipid-based NPs, including liposomes, represent the most common nanocarrier platform currently used at the clinical stage for liver fibrosis treatment [21–23].

### Nanocarrier–liver interactions

**Figure 2:**
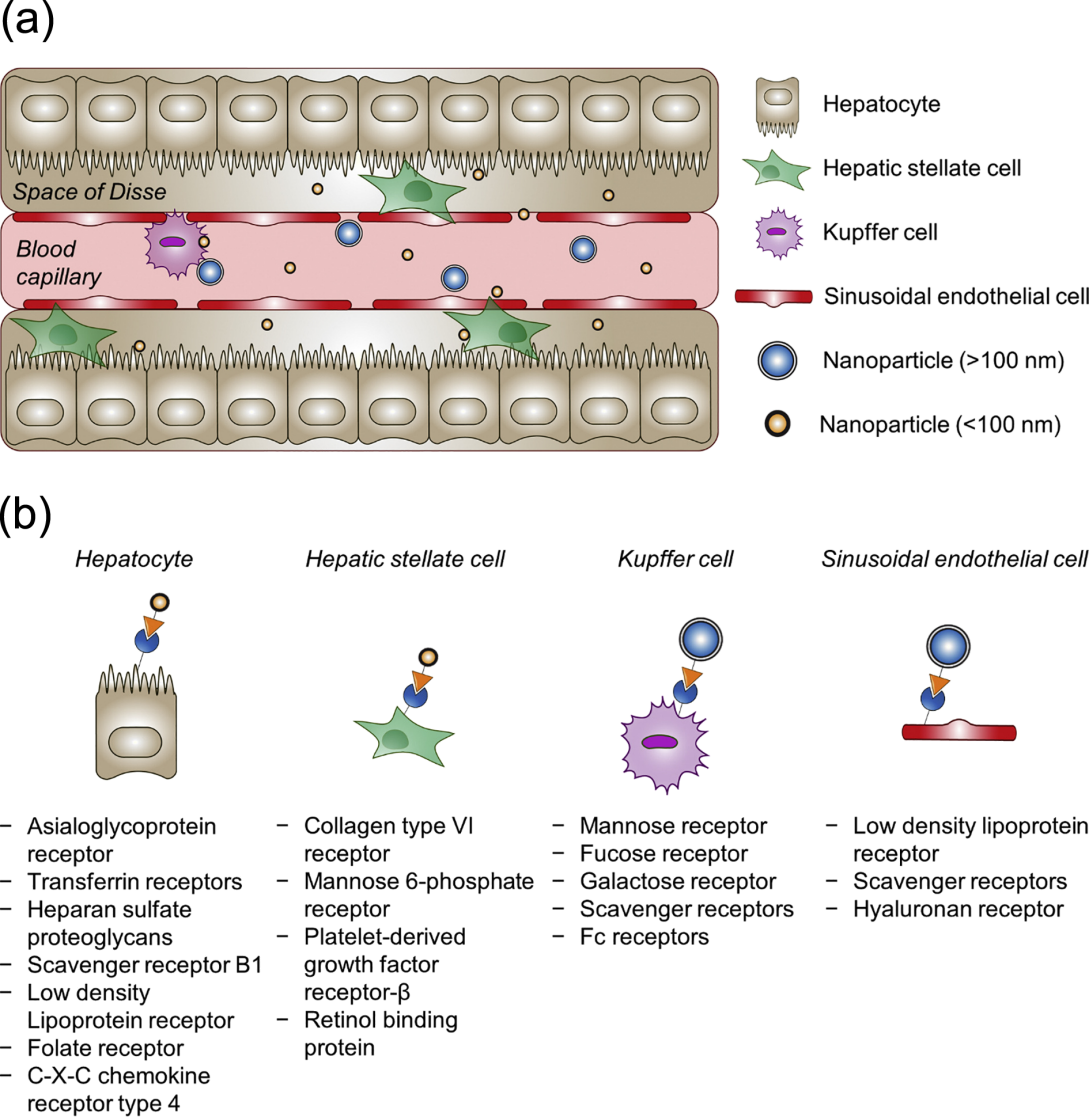
Schematic illustration of (a) passive and (b) active targeting strategies for liver delivery systems. (a) Size-dependent accumulation of nanocarriers in liver sinusoids for passive targeting. (b) List of overexpressed receptors on specific populations of liver cells for active targeting. Figure 2a,b was reprinted from [29], Advanced Drug Delivery Reviews, vol. 154-155, by R. Böttger et al., “Lipid-based nanoparticle technologies for liver targeting“, pages 79-101, Copyright (2020), with permission from Elsevier. This content is not subject to CC BY 4.0.

The accumulation of any type of NPs in the liver is generally accomplished because of the central role of the liver itself as a main metabolic and excretory organ in the body. The presence of fenestrations in the layers of liver sinusoidal endothelial cells (LSECs) and the absence of the impermeable basal lamina allow for rapid accumulation of NPs in the liver through passive targeting [24]. Complementing certain anatomic or pathophysiological features of the target organ, such passive accumulation also relies on nanoparticle properties including size, shape, surface charge, and hydrophilicity [25]. For instance, the passive liver targeting strategy highly depends on the size of nanocarriers as the endothelial fenestrations of liver sinusoids span approximately 50–200 nm in diameter (Figure 2a). In their study, Hirn and co-workers revealed that 50% of small gold NPs (around 1.4 nm) were accumulated in the liver after systemic administration. As the size was increased into 200 nm, the fraction of gold NPs accumulated in the liver was further increased to 99% [26]. The fact that around 80–90% of all macrophage population resides in the liver also contributes to the passive accumulation of NPs in the liver [27–28].

The interaction of the nanocarriers with various types of cells is size-dependent [30]. Nanocarriers with a particle size bigger than 100 nm could be taken up by LSECs and Kupffer cells through endocytosis. With the increase of particle size, the uptake of nanocarriers by Kupffer cells is enhanced, leading to rapid accumulation of the nanocarriers in the liver with short blood circulation time [31]. In contrast, nanocarriers with a particle size smaller than 100 nm could avoid a capture by Kupﬀer cells. These nanocarriers then diﬀuse out of the sinusoids through the dynamic endothelial fenestrations to reach HSCs in the perisinusoidal space or even hepatocytes [24,29]. Smaller nanocarriers (10–20 nm) can be taken up rapidly by hepatocytes [32].

Besides the size of the administered nanocarriers, surface properties also play an important role in dictating hepatobiliary clearance in vivo. For example, positively charged mesoporous silica NPs (MSNPs) underwent significant uptake by hepatocytes, while MSNPs with negative charges were rapidly internalized by Kupffer cells in liver sinusoids [33]. The negative charge of the nanocarriers could facilitate efficient binding to the scavenger receptors on the surface of Kupffer cells and LSECs, leading to the improved uptake by these cell types [34–35].

Another intrinsic property that dictates the uptake pathway of the nanocarriers by liver cells is hydrophilicity/hydrophobicity. Increased hydrophilicity via decoration with polyethylene glycol (PEG) may minimalize the uptake of nanocarriers by Kupffer cells, and such PEGylated nanocarriers are likely taken up by hepatocytes [36–39]. Besides augmenting hydrophilicity through PEGylation, stealth properties could be endowed to the nanocarriers through a serum albumin corona to escape Kupffer cells’ clearance, facilitating direct uptake in hepatocytes [40]. Hydrophobic nanocarriers are rapidly cleared from the systemic circulation by Kupffer cells [33].

In the pathophysiological condition of hepatocellular carcinoma, microvascular density and permeability are increased because of angiogenesis within the tumor microenvironment [41]. The fenestrae of the liver endothelial cells increase to approximately 400–600 nm, often accompanied with impaired lymphatic drainage, leading to the EPR effect [42]. In contrast, the excessive production of ECM by the activated HSCs in liver fibrosis resulted in a loss of the fenestrae, hindering plasma to reach the perisinusoidal space [43]. As the disease progresses, the reduced blood flow and the blockage of portal ﬂow through the liver could diminish the efficiency of drug delivery.

### Nanoencapsulation as passive targeting strategy to the liver

To endow passive targeting, specific nanocarriers with extended blood circulation profile are favorable to achieve an improvement in the bioavailability of the active pharmaceutical ingredients (APIs) and an increased accumulation at the target site. Nanoencapsulation of APIs through modified nanocarriers could enhance their bioavailability by altering the pharmacokinetics as well as by protecting the unstable cargo against environmental factors [44]. Various potent antifibrosis substances from synthetic and herbal compounds suffer from limited solubility and lack of stability, resulting in poor bioavailability.

Regarding synthetic substances, Kurniawan and co-workers encapsulated the potent inhibitor R406 to inhibit spleen tyrosine kinase in inflammatory macrophages using poly(lactic-*co*-glycolic acid) (PLGA) NPs (R406-PLGA) [45]. PLGA was used as polymeric platform as it is an FDA-approved biodegradable polymer. The R406-PLGA NPs (particle size of 159.7 nm) showed a significant downregulation of major inflammatory markers (CCL2, IL-1α, and IL-6) in vitro in murine bone marrow-derived macrophages. In an in vivo experiment using a methionine and choline-deficient (MCD) mouse model, the efficient intrahepatic delivery of R406-PLGA NPs ameliorated liver inflammation, fibrosis, and hepatic steatosis, probably because of improved pharmacokinetics and bioavailability of R406. Despite its favorable toxicity profile, only 19 drug formulations based on PLGA have been approved by the FDA up to 2019 [46]. They consist of PLGA microparticles, solid implants, and in situ gels; none of them is a PLGA NP formulation. This fact indicates that there are some challenges, including poor drug entrapment efficiency and drug release kinetics from PLGA nanoformulations [47].

Regarding plant-derived compounds, curcumin is an ideal representative of phytocompounds with antifibrosis activity. Despite a large volume of published reports on curcumin, curcumin’s major constraints in clinical trials include short biological half-life in plasma and low bioavailability. To solve these limitations, nanoencapsulation of curcumin has been developed, and some of these formulas are undergoing clinical trial evaluation [48–50]. By exploiting this technique, the oral bioavailability of encapsulated curcumin could be improved at least ninefold compared to curcumin administered with piperine as an absorption enhancer [51].

The therapeutic potential of curcumin using nanoformulations was reviewed by several researchers, summarizing recent curcumin encapsulation works on various NP platforms (liposomes, solid lipid NPs, micelles, and polymeric NPs) [52–53]. For example, polymeric nanoparticle-encapsulated curcumin (NanoCurc™) could ameliorate CCl_4_-induced hepatic injury and fibrosis through reduction of pro-inflammatory cytokines [54]. The polymer platform of NanoCurc™ consists of *N*-isopropylacrylamide, vinylpyrrolidone, and acrylic acid and was selected because of its capability to dissolve a broad range of poorly water-soluble drugs. As this polymeric platform could deliver substantial amounts of curcumin to the liver, a significant reduction in in vivo CCl_4_-induced hepatocellular injury could be observed. The toxicity data also shows that NanoCurc™ essentially exhibits no toxicity upon daily systemic administration through the intraperitoneal route in mice [54].

Another polymeric platform to improve the bioavailability of curcumin was developed through simple nanoemulsification using biodegradable polylactide–poly(ethylene glycol) (PLA-PEG) copolymer NPs [55]. Besides reversing the elevation of plasma enzyme activity of aspartate transaminase (ALT) and alanine transaminase (AST), the orally administered curcumin loaded PLA-PEG NPs successfully improved the in vivo structure of the liver and reduced microvesicular steatosis, congestion of erythrocytes, and the infiltration of inflammatory cells. Both PLA and PEG have been authorized by the FDA. The low molecular weight of PLA is preferable to construct nanocarriers because of its relatively fast degradation rate with non-toxic degradation products (H_2_O and CO_2_) [56].

**Figure 3:**
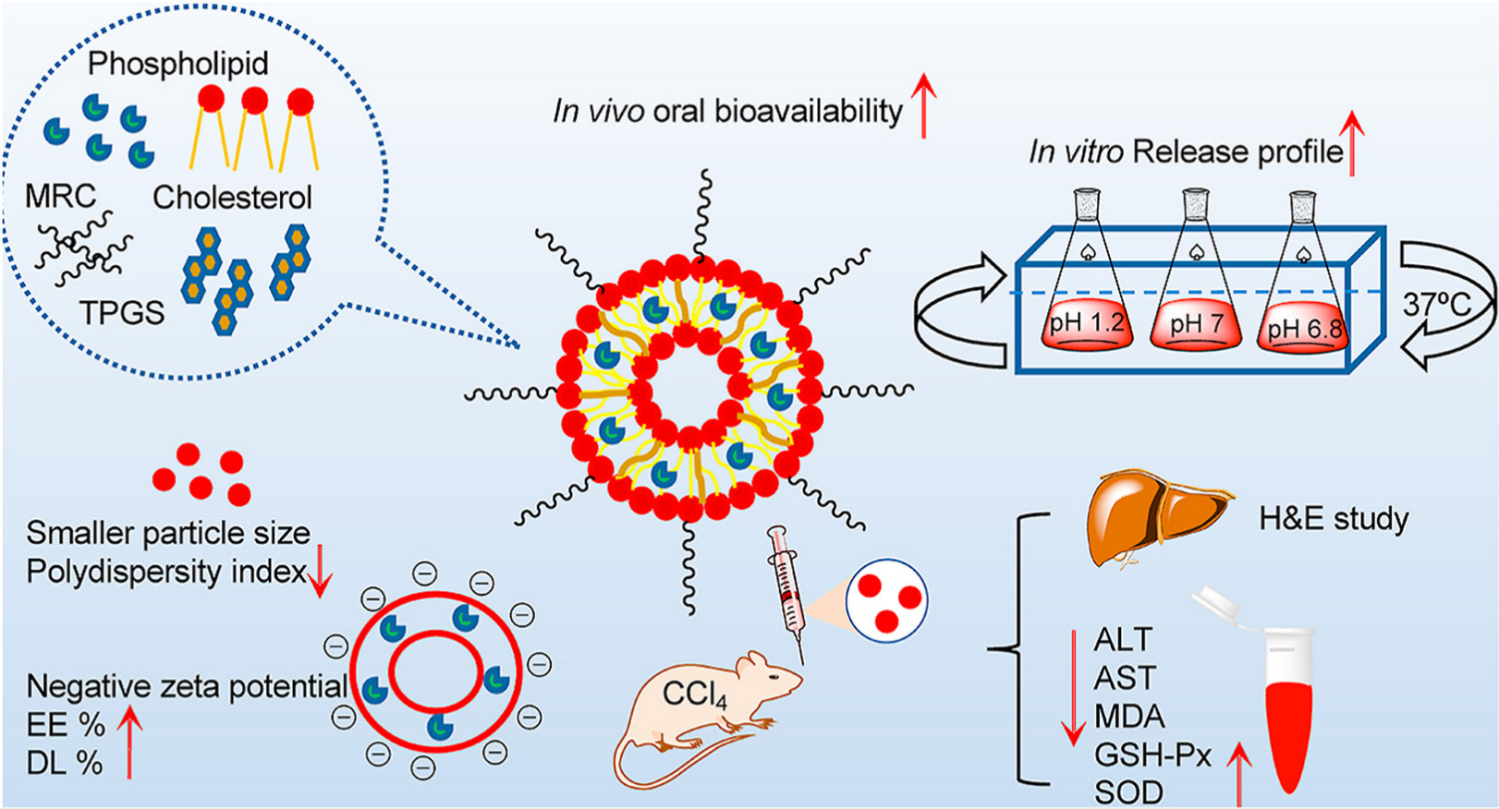
Schematic illustration of non-targeted pro-liposome myricetin nanocarriers modified with vitamin E-TPGS on its surface. Figure 3 was reprinted from [57], Journal of Drug Delivery Science and Technology, vol. 66, by Y. Thant et al., “TPGS conjugated pro-liposomal nanodrug delivery system potentiate the antioxidant and hepatoprotective activity of Myricetin“, article no. 102808, Copyright (2021), with permission from Elsevier. This content is not subject to CC BY 4.0.

Regarding liposomal platforms, Thant and co-workers encapsulated the antifibrosis compound myricetin in pro-liposome nanocarriers to improve its solubility, stability, and low oral bioavailability [57]. As illustrated in Figure 3, the surface modification of pro-liposome with ᴅ-α-tocopheryl polyethylene glycol 1000 succinate (vitamin E-TPGS) enhanced the stability and passive targeting effect of the pro-liposomal drug delivery system. The in vivo pharmacological activity of the pro-liposomes displayed a 7.2-fold increased oral bioavailability of myricetin, leading to remarkably decreased levels of ALT, AST, and the lipid peroxidation marker (MDA), while enhancing the antioxidant defense mechanism. Besides providing the containment for the active substance, the nanostructured lipid nanocarriers could be utilized for targeted delivery without conjugating any specific ligand. Composed of natural phospholipids, liposomes are generally considered to be pharmacologically inactive with minimal toxicity [58]. The increasing trend of liposomal formulations translated into clinical applications highlights the potency of liposomes as nanocarriers.

Wang and co-workers utilized a phosphatidylserine for constructing curcumin-loaded lipid-based nanocarriers [59]. Phosphatidylserines are anionic phospholipid components of cell membranes, which act as a specific recognition signal for phagocytosis of apoptotic cells by macrophages [60]. Phosphatidylserine modification allows for efficient delivery of active substances to specific sites enriched with macrophages [61]. The encapsulation of curcumin in the phosphatidylserine nanocarrier improved its in vivo retention time, while free curcumin was quickly cleared from the body. As a consequence of the altered pharmacokinetics of the curcumin nanocarrier, the accumulation of curcumin in the liver was also enhanced, confirming its liver-targeting properties. Targeting of the curcumin nanocarrier, therefore, significantly improved the in vivo therapeutic outcome of curcumin, as shown by reduction of liver fibrosis biomarkers, proinflammatory cytokine levels in the serum, and liver collagen deposition.

Besides providing a carrier for active substances, some NPs, particularly inorganic NPs, can act as therapeutic agent because of their intrinsic pharmacological properties, contributing to the amelioration of liver fibrosis [62]. For example, Hamza and co-workers exploited the vital micronutrient selenium to form SeNPs for their antioxidant, antibacterial, and anti-inflammation properties [63]. The combined administration of SeNPs and vitamin E resulted in an in vivo reduction of hepatic enzyme activity induced by acrylamide while also improving the lipid profile and histological hepatic tissues.

In addition, zinc oxide NPs displayed hepato-protective effects in dimethylnitrosamine-induced liver injury, which may be due to selective toxicity to the proliferating tissue, including adenomatous islands formed in the liver [64]. Peng and co-workers successfully elucidated the hepato-protective effect of TiO_2_ NPs and SiO_2_ NPs [65]. The effects of TiO_2_ NPs, with diameters around 20 and 200 nm, and SiO_2_ NPs on proliferation, fibrosis, adhesion, and migration of LX-2 cells as a model of HSC activation were studied. The results show that the internalization of both TiO_2_ NPs and SiO_2_ NPs suppressed classical outcomes of cellular fibrosis, including the reduced expression of collagen I and alpha smooth muscle actin. These NPs also contributed to proteolytic breakdown of collagen by up-regulation of matrix metalloproteinases (MMPs) and down-regulation of tissue inhibitors of MMPs (TIMPs). TiO_2_ NPs also could induce enhanced leakiness and drug permeability in primary human HSECs during liver fibrosis and occlusion [66]. The exposure of TiO_2_ NPs in vitro resulted in the formation of large gaps between the cells without significant effects on cell viability and no significant release of oxidative stress. This strategy may be exploited for co-delivering antifibrosis substances by increasing the number of drugs crossing through HSECs and, subsequently, improving the therapeutic outcomes. Despite the significant recognition for treating liver fibrosis, the potency of inorganic NPs may be limited by their non-biodegradability and potential toxicity. Further toxicity studies are absolutely needed to assure the safety of this particular fibrosis treatment.

### Active liver targeting

For active liver targeting, cells including HSCs, macrophages, LSECs, and hepatocytes are the main therapeutic targets because these cells contribute to liver fibrosis progression. Among the various molecular mechanisms causing liver ﬁbrosis, activation of HSCs is considered a central link. This is because of the dominant contribution of activated HSCs in ECM production, making HSC activation a preferred target for antiﬁbrotic therapy [67]. In any of the aforementioned liver cell types, there is a particular receptor that could be targeted to specifically deliver antifibrotic drugs, as illustrated in Figure 2b. On the surface of HSCs, the mannose 6-phosphate receptor (M6PR) is probably the most prominent receptor for targeting HSCs. This particular molecule is overexpressed in activated HSCs and strongly related to the transformation of HSCs into matrix-producing myoﬁbroblasts during liver fibrinogenesis [68–69]. The HSC-mediated antifibrotic drugs work through several mechanisms, including inhibition of fibrogenesis (ECM synthesis), inhibition of HSC proliferation, inhibition of profibrogenic cytokine and growth factors secretion, and induction of ECM degradation [70].

A summary of liver-targeted delivery systems is shown in Table 1. Passive and active delivery strategies were combined by Luo and co-workers, who prepared silibinin–human serum albumin nanocrystals [71]. The prepared nanocrystals displayed enhanced solubility and in vivo bioavailability of silibinin, which is known for its low solubility and low permeability. The relatively small silibinin nanocrystals (ca. 60 nm) also could passively extravasate through pores of ca. 100 nm diameter in LSECs before targeting activated HSCs. The presence of HSA on the surface of the nanocrystals may facilitate active targeting to activated HSCs via secreted protein acidic and rich in cysteine (SPRAC)-mediated endocytosis. It was reported that the activated HSCs overexpressed SPRAC, which is known as classic albumin binding protein. This protein is not available in hepatocytes, thus enabling specific targeting to activated HSCs in liver fibrosis [72].

**Table 1:** Various active-targeting nanocarriers for liver fibrosis treatment.

Liver cell type	Target receptor	Ligand	Active substances	Nanocarriers	Ref.

HSCs	mannose 6-phosphate receptor	mannose 6-phosphate	matrine	solid lipid NPs	[73]
		mannose 6-phosphate	*Pcbp2* siRNA	cholesteryl peptide-based micelle	[74]

	retinol binding protein	vitamin A	siCol1α1 and siTIMP-1 siRNAs	lipid NPs	[75]
		vitamin A	Rho/Rho-associated protein kinase (ROCK) inhibitor	liposome	[76]
		retinoic acid	galangin	Eudragit^®^ RS100, Eud RS100 NPs	[77]

	retinol binding protein & sigma-1 receptor	vitamin A and aminoethylanisamide	siRNA against IL11	PEG-PLGA NPs & cationic lipid-like molecule	[78]
	folate receptor alpha	folic acid	camptothecin	micelle	[79]
	fibroblast growth factor receptor	fibroblast growth factor 2	fibroblast growth factor 2	superparamagnetic iron oxide NPs	[80]
	relaxin family peptide receptor 1 (RXFP1)	relaxin	relaxin	superparamagnetic iron oxide NPs	[81]

Hepatocytes	asialoglycoprotein receptor	trivalent *N*-acetyl-ᴅ-galactosamine (GalNAc)	siPLK1	lipid NPs	[82]
		lactose	selastrol	albumin nanoparticles	[83]
		galactose	resveratrol	starch-lysozyme nanocarriers	[84]
		galactose	—	dendrimer	[85]

LSECs	hyaluronic receptor	hyaluronic acid	simvastatin	lipid NPs	[86]
	mannose receptor	mannan	simvastatin	PLGA-PEG NPs	[87]
	stabilin receptors	ApoB peptide	rapamycin and curcumin	ovalbumin NPs	[88]

Kupffer cells	mannose/fucose receptors	4-aminophenyl-α-ᴅ-mannopyranoside and 4-aminophenyl-β-ʟ-fucopyranoside	—	liposome	[89]
	scavenger receptor CD163	anti-CD163 monoclonal antibody	vitamin D_3_	lipid NPs	[90]

Tan and co-workers also constructed albumin–mannose 6-phosphate-modified solid lipid NPs to deliver matrine to HSCs [73]. The active substance matrine was first loaded into the solid lipid NPs using the microemulsion-probe ultrasound method, while mannose 6-phosphate was conjugated to albumin. The mannose 6-phosphate-conjugated albumin was then decorated onto the surface of matrine-loaded solid lipid NPs, and its HSC-targeting efficiency was evaluated in vitro and in vivo. In the vivo experiment, these carriers showed specific accumulation in CCl_4_-induced liver fibrosis in mice as proved by the reduction of fibrotic biomarker levels. The nanocarrier also inhibited the activation of HSCs and slowed down the progression of liver fibrosis as shown by the low inflammatory infiltration and the disruption of liver structure and collagen deposition. The enhanced therapeutic actions of matrine-loaded albumin–mannose 6-phosphate-modified solid lipid NPs may be due to an increased plasma concentration facilitated by albumin decoration, leading to passive accumulation of the nanocarriers. The matrine-loaded nanocarrier also displayed active targeting as shown by higher accumulation of matrine nanocarrier in the liver, while free matrine and matrine nanocarrier without targeting ligand showed relatively low liver accumulation.

Yin and co-workers developed a cholesteryl peptide-based micelle nanocomplex for delivering *Pcbp2* siRNA as gene-silencing agent [74]. The surface of the nanocarrier was modified with a dimeric IGF2R peptide as a M6PR-targeting ligand of the activated HSCs. The use of cholesteryl peptide to construct the nanocarrier facilitated in vitro cellular uptake in a time-dependent manner. Ultimately, the nanocomplex showed excellent in vitro gene-silencing activities, that is, approximately 80–85% of the *Pcbp2* mRNA expression was inhibited in activated HSCs-T6 cells after gene transfection for 24 h. The in vivo biodistribution showed that the nanocomplex specifically accumulated in fibrotic liver tissue in rats with CCl_4_-induced liver fibrosis, while the unmodified nanocomplex displayed low accumulation because of rapid clearance from the body.

**Figure 4:**
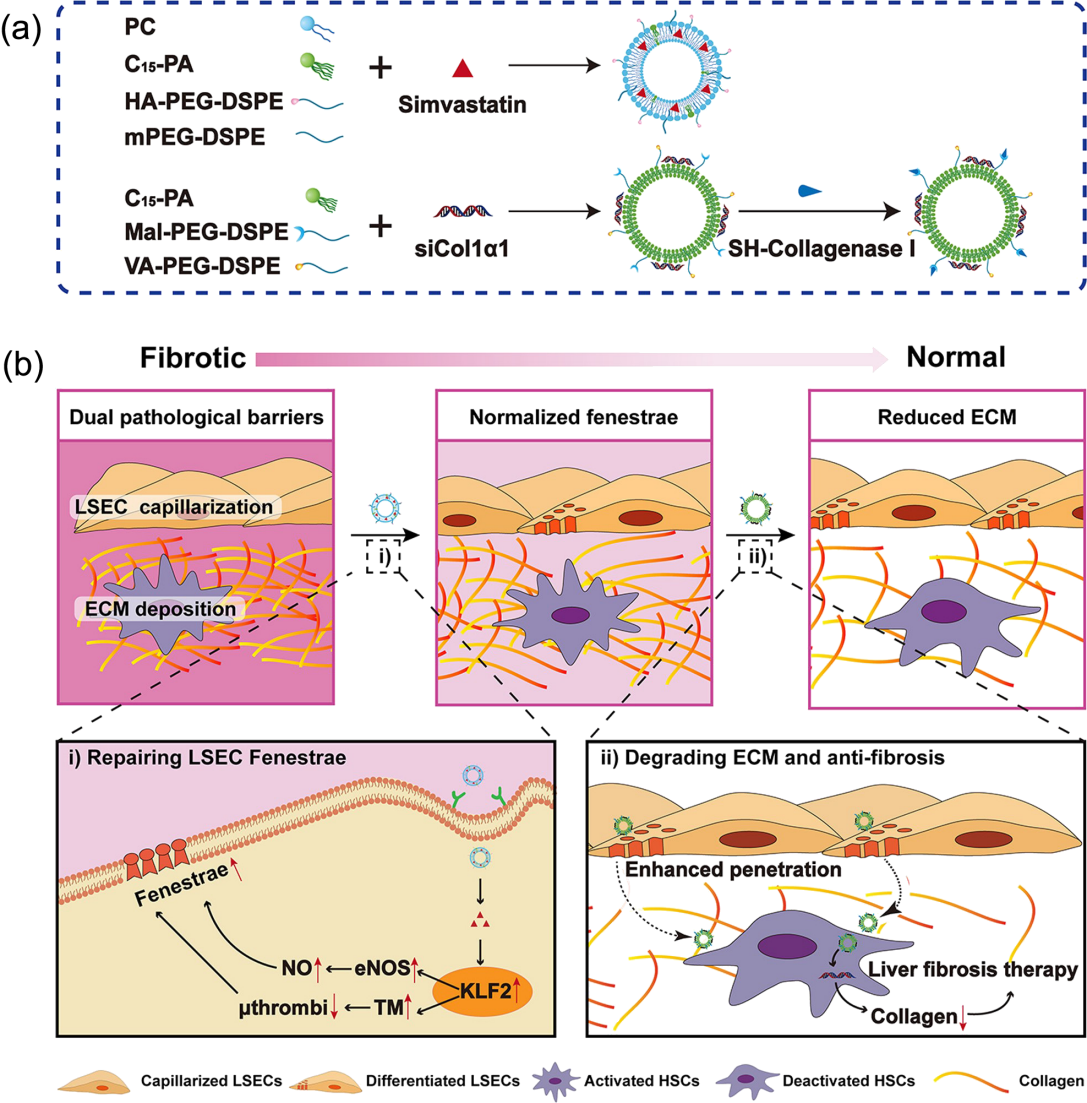
(a) Preparation of a dual-NP co-delivery system of HA-NPs/SMV and CV-NPs/siCol1α1 to target LSECs and HSCs, respectively, and (b) its working mechanism in vivo to reverse liver fibrosis. Figure 4a,b was reprinted with permission from [86], Copyright 2022 American Chemical Society. This content is not subject to CC BY 4.0.

Another strategy to deliver antifibrotic substances to HSCs is using vitamin A to target retinol-binding protein. This is due to the fact that HSCs account for 80% of vitamin A in the liver because of the overexpression of the retinol binding protein on the HSCs [91]. Qiao and co-workers used the concept to deliver siRNAs to HSCs using lipid NPs as platform, as illustrated in Figure 4a [75]. In liver fibrosis, siCol1α1 and siTIMP-1 siRNAs were used to inhibit collagen synthesis and to promote collagen degradation, respectively. The spherical lipid NPs with a mean particle size of 140 ± 0.12 nm and negative zeta potential (−12.9 mV) were constructed from amphiphilic cationic hyperbranched lipoids for siRNA complexation and cholesterol–polyethylene glycol–vitamin A as a helper lipoid. In the in vitro evaluation, the nanocarrier showed enhanced cellular uptake in HSCs-T6 cells, nine times higher than that in macrophages, displaying specific targeting to HSCs that could avoid phagocytosis by macrophages. These cellular uptake results accordingly affected the in vitro gene silencing activity as shown in decreased expression of Col1α1 and TIMP-1 after administering the nanocarriers. The in vivo cellular localization of siRNA-VLNPs in the liver tissue was evaluated in CCl_4_-treated mice. The result shows that the vitamin A-modified nanocarriers co-localized in HSCs, highlighting the success of targeted delivery. The therapeutic activity evaluation also revealed consistent results, showing decreased liver fibrosis in histological images, with low collagen accumulation and low serum biomarker level (i.e., AST and ALT).

To further improve the therapeutic outcome of the liver-targeted nanocarriers, recently, Zhang and co-workers developed a dual-nanoparticle co-delivery system for targeting LSECs and HSCs [86]. Two types of DSPE-PEG NPs were prepared, and each of them was decorated with either hyaluronic acid or a combination of collagenase I and vitamin A, as shown in Figure 4b. Simvastatin-encapsulated hyaluronic-modified NPs (HA-NPs/SMV) were designed to target capillarized LSECs. The collagenase I and vitamin A-modified NPs entrapped siCol1α1 (CV-NPs/siCol1α1) and were constructed to inhibit collagen generation and HSC activation, as illustrated in Figure 4b. These two NPs works sequentially; the rapid release of SMV from SMV HA-NPs/SMV exerted a fenestrae-repairing function of LSECs, and the vanished fenestrae in LSECs allowed more CV-NPs/siCol1α1 to enter the perisinusoidal space to degrade deposited collagen and finally to achieve higher accumulation in activated HSCs.

## Conclusion

This review summarizes recent updates in applications of nanomedicine for the treatment of liver fibrosis. Conventional drugs need a specified targeted delivery carrier to deal with unfavorable properties of the drugs themselves and to overcome physiological barriers. Targeted carriers containing multiple drugs may be a future research direction of liver fibrosis treatment since the disease progression involves multiple signal pathways, which may limit the efficacy of single-drug therapies. The combination of therapeutic agents in a single nanocarrier has been a primary goal particularly for nanomedicine-based cancer immunotherapy, allowing for both suppression of tumor growth and inhibition of metastatic spread. Recent research trends on active targeting strategies for hepatic fibrosis still focus on exploiting HSCs as target because the activation of these cells is the central event underlying liver fibrosis. Considering the involvement of multiple cell types on the exacerbation of hepatic fibrosis, more studies targeting other liver cells should be carried out using various ligands.

One needs to take into account that the incorporation of a broad range of therapeutic agents in a single nanocarrier makes the formulations more complex. Therefore, adequate concerns about standardized protocols and trans-disciplinary characterization strategies of the nanoproducts product should be addressed. A comprehensive set of characterization procedures using state-of-the-art nanomedicine manufacturing facilities allows for continuous monitoring of the production steps, which is required to maintain the consistency of nanocarriers,

Despite its promises, the long-term hepatotoxicity of NPs should be carefully reviewed as 30–99% of the administered NPs will be accumulated and sequestered in the liver [32]. Liver is the main organ of metabolic clearance of most drugs, and liver fibrosis definitely disturbs its clearance function. The potential risk that the exposure of NPs may increase pathological damage to the liver should be given appropriate attention. Thus, complete understanding of NP toxicity during exposure to the cells is needed to provide information on the NPs regarding safety profiles and long-term effects on liver and other organs. Currently, the research on liver toxicity of the particular nanocarriers is limited compared to work on designing nanocarriers for liver fibrosis and elucidating their work mechanism. Also, there are some difficulties to reevaluate the published toxicity reports and to compare them among each other because of non-standardized protocols used to evaluate the toxicity, leading to conflicting comparison results. To improve the validity of the toxicity profile of the NPs, it is important to ensure the accuracy, reliability, and reproducibility of the experimental data. In the end, we expect that the obtained toxicity data could be comprehensively compared with other toxicity reports.

## Data Availability

Data sharing is not applicable as no new data was generated or analyzed in this study.
